# Provider connectedness and communication patterns: extending continuity of care in the context of the circle of care

**DOI:** 10.1186/1472-6963-13-309

**Published:** 2013-08-14

**Authors:** Morgan Price, Francis Y Lau

**Affiliations:** 1 School of Health Information Science, University of Victoria, 3800 Finnerty Road Victoria, V8P 5C2 Victoria, British Columbia, Canada; 2 Department of Family Practice, University of British Columbia, Vancouver, Canada; 3 Medical Science Building, University of Victoria, PO Box 1700, STN CSC, Victoria BC V8W 2Y2 Canada

**Keywords:** Continuity of care, Circle of care, Quality of care, Communication, Systems thinking

## Abstract

**Background:**

Continuity is an important aspect of quality of care, especially for complex patients in the community. We explored provider perceptions of continuity through a system’s lens. The circle of care was used as the system.

**Methods:**

Soft systems methodology was used to understand and improve continuity for end of life patients in two communities. Participants: Physicians, nurses, pharmacists in two communities in British Columbia, involved in end of life care. Two debates/discussion groups were completed after the interviews and initial analysis to confirm findings. Interview recordings were qualitatively analyzed to extract components and enablers of continuity.

**Results:**

32 provider interviews were completed. Findings from this study support the three types of continuity described by Haggerty and Reid (information, management, and relationship continuity). This work extends their model by adding features of the circle of care that influence and enable continuity: *Provider Connectedness* the sense of knowing and trust between providers who share care of a patient; a set of ten *communication patterns* that are used to support continuity across the circle of care; and *environmental factors* outside the circle that can indirectly influence continuity.

**Conclusions:**

We present an extended model of continuity of care. The components in the model can support health planners consider how health care is organized to promote continuity and by researchers when considering future continuity research.

## Background

### Continuity of care

Continuity of care is a key component of quality of care [1,2] that becomes increasingly important for complex and vulnerable patients [3,4], including elderly patients [5], patients at end of life [2,6], those with chronic conditions [7,8], and those who transition through the greater healthcare system [9-11]. Continuity is central to many health disciplines, including family medicine [12,13] and nursing [14,15] and is an important national issue in several countries [16-18]. Continuity has been defined in many ways [13]. Several groups have developed models to describe continuity of care [14,15,19-22] and there are multiple factors that are correlated with continuity [23].

Haggerty and Reid systematically reviewed continuity across disciplines and they defined continuity of care as: the perceived coordination of care for a single patient over time [19]. Continuity includes how the discrete care events interrelate and how they are effectively communicated and managed amongst all members of the care team. Their model consists of three types of continuity: information continuity, management continuity and relationship continuity. They acknowledge that continuity can be perceived differently by the patient and by providers [19]. Patients often assume continuity occurs until they experience gaps [24]. Gaps in continuity occur when there is a lack of coordination between providers and between providers and the patient. Communication is the “glue” between members of the care teams and care settings to support continuity [17].

### Soft systems methodology

Complex or wicked problems [25,26], such as improving continuity of care, are suited to a systems thinking approach [27]. Soft Systems Methodology (SSM) [27-29] has been developed as a method to explore complex organizational and cross-organizational systems. Thus, SSM was considered well suited to exploring continuity as a systems challenge.

SSM explores a challenge or problem with a range of stakeholders through the development of relevant models of the problem that are then compared to the real world situations and experience to debate on potential improvements. It makes these comparisons largely through visual *rich pictures* and *conceptual models* and uses these to focus debates on gaps and challenges. Through these debates, multiple stakeholders can express contrasting views and collectively explore the problem to come to a common understanding and the potential improvements. SSM been used successfully to explore a range of issues [30], including challenges in the healthcare system. For example, SSM has been used to improve outpatient services [31,32] and supported early work in the creation of information systems for the National Health Service in England [33].

Systems are conceptualizations of an interrelated whole [34] and they can be complex and adaptive [35,36]. Selecting an appropriate system is key to systems thinking. For understanding aspects of continuity, we propose the circle of care is an appropriate system.

### Circle of care: the patient’s healthcare system

We define the *circle of care* as an individual patient’s healthcare system. The circle of care is a soft system that consists of the patient, providers, other agents, and the information repositories (paper and electronic) related to that patient. It is self-organizing, can span organizations, and changes based on the needs of the patient and availability of resources.

In this paper, we have used the circle of care to aid our understanding of continuity of care and have focused on provider experience of continuity as they may be more aware of continuity challenges that patients are not. This paper presents an extended model for continuity, where continuity of care is a property of a complex system: the patient’s circle of care.

## Methods

This extended model of continuity of care was developed as part of a larger, mixed-method, action research study that used an adapted SSM approach to discover improvements in continuity for end of life patients. The study was performed in 2009 in two communities in British Columbia, Canada [37]. This study conforms to the qualitative guidelines as set out by BioMedCentral, the specific methods are described below.

### Participants and recruitment

Participants included providers in the two study communities who were actively involved in providing end of life care. Participants were recruited in a snowball method, starting with family physicians. Family physicians provided the majority of longitudinal care in the region and thus were a logical group to start interviewing. Six family physicians were recruited for the first round of interviews (three in each community). Based on findings from the first round of interviews (interviews described below) additional participants were recruited. Additional interviews with new roles were considered when participants described clear communication activities. That is, additional roles were involved in directly maintaining continuity (e.g. family physician and home care nurse would speak directly about a patient’s pain management). Roles for this study were more detailed than professional role. For example, roles included: home and community care nurse, hospice nurse, community palliative care nurse instead of just nurse. The snowball method occurred independently in each community and allowed the circles to be defined through the research.

Two to four individual participants for each role in each community were recruited through regional leaders (e.g. hospice director, regional director of primary care). Recruitment letters were sent out through email by the recruiter and the potential participants contacted the researcher. Recruitment in this manner was required by ethics as some participants were staff of the regional health authority. Information sessions on the study were also provided to various groups during recruitment (e.g. to home and community care). End of life patients were excluded from interviews due to the expected challenges recruiting those patients at a difficult time of their lives. This also focused the research on understanding continuity from the providers perspective, which can be different than the patient [24].

### Data collection: interviews

**Figure 1:**
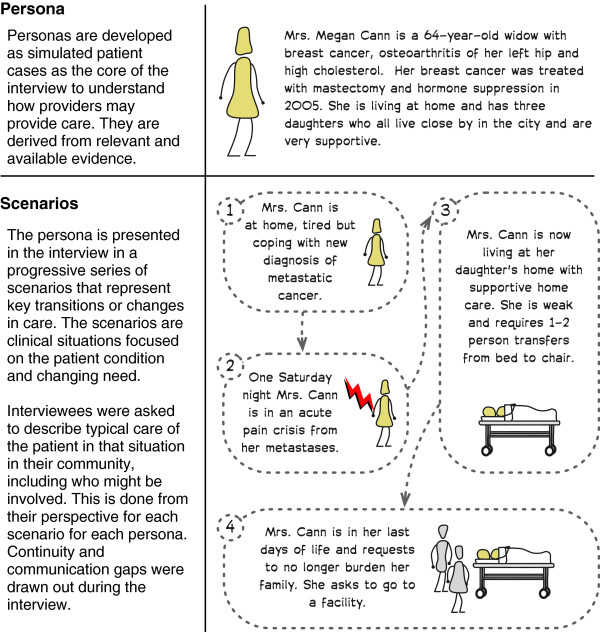
**The persona Mrs. Cann that was one of the two personas used to structure the participant interviews.** Four scenarios were presented to the participants and they provided details on how they perceived care was delivered and continuity achieved in each of the scenarios.

Participant interviews were one on one, semi-structured interviews that lasted 1-2 hours each. Two simulated end of life patient cases or *personas* (see example persona, Mrs. Cann, in Figure 1) were used to structure the interviews. The personas were developed from a review of end of life care in British Columbia [38] to illustrate common features of end of life care. One persona, Mrs. Cann, was a patient with breast cancer. The other, Mr. Hart, developed congestive heart failure. Four distinct scenarios were developed for each persona. These scenarios described transitions/changes for the persona over the last year of life, such as: needing acute pain management, moving to long-term care, and needing inpatient care.

In the interviews, providers described care activities, roles they saw were involved in care, the communication activities, and typical challenges in continuity in each scenario (see Table 1 for example interview questions). Participants signed consent and interviews were audio recorded. Interviews were completed when saturation was reached. Saturation was defined as: no new provider roles identified that had active roles in maintaining continuity of care. Interview participants were recruited at the end of the interview to participate in a discussion group to review and debate the findings.

**Table 1:** Structure and example questions used for the provider interviews

**Interview topic**	**Description/example questions**
Participant Information	• What is your Profession or role in supporting end of life care?
	• How many years have you been in practice?
	• Gender: M/F
	• Please briefly describe your practice?
Persona Information	*Interviewer provides information about the patient case and then about each of the specific scenarios. This repeats for each of the two cases, with questions below being asked for each scenario.*
Scenario Questions (For each scenario of each persona)	• Who would have engaged you in this patient’s care (e.g. another provider, patient themselves)?
	• How would they have communicated to you the need for your involvement?
	• Where would you gather information about this patient (e.g. do you have a referral letter, chart, patient themselves)?
	• What information do you often need that is missing for patients like this?
	• Would you access information from another electronic record/electronic system?
	• From your experience, who else would be engaged in < *PERSONA NAME* > 's care at this point? (Please describe the various provider roles you would expect to be involved)
Communication Questions	The questions below would be asked or each role described involved in the patients care above:
	• What role are they playing in the care of our patient at this point?
	• How do you communicate with this provider?
	• (Describe what you communicate)
	• When does this happen?
	• How do they communicate with you?
	• (Describe what they communicate)
	• Which methods of communication are most important to you to ensure Continuity of Care?
	• If you do not communicate directly with this provider, should you?
	○ If yes, please tell me why it would be important?
	○ If No, please tell me why would it not be important?
	• How can you improve Continuity of Care with this provider?
	• How could the provider improve Continuity of Care with you?

The interviews were structured around two patient personas that were used to highlight communication activities and gaps in continuity.

### Debates/discussion groups

The findings from the interviews were shared in two, two-hour multidisciplinary debates or discussion groups (one for each community). The goals of the discussion groups were to: share findings, review the extended model of continuity of care, and review recommendations for improving continuity of care. All debate participants had participated in interviews and signed a second consent at the start of the debate. The personas were re-used from the interviews to trigger debate and discussion on applicability of suggested improvements. Revised personas, which had different details developed for each community based on the interviews, were used to illustrate challenges in continuity and suggested improvements. The personas were used transcend differences in opinion on the expected impact of specific improvements. Improvements needed to support both personas in both communities in at least one key scenario with a gap in continuity of care to be considered. Feedback from the multi-stakeholder debate/discussion groups were used to revise the elements of the extended continuity of care model.

### Data analysis

Interview and debate recordings were analyzed iteratively through the study. Deductive and inductive theming was used to analyze the recordings. Deductive theming included using the three previously defined types of continuity: information, management, and relationship continuity [19]. Inductive theming was used to discover new aspects of continuity.

Communication activities related to maintaining continuity of care were captured and any example forms were collected. These were coded and themed together based on intention of the communication activity. Thus, a set of communication patterns was developed based on the various intentions related to continuity of care.

One researcher (MP) completed all interviews and analyzed the recordings. The second author (FL) and a supervising committee regularly reviewed findings. Ethics was received from the Joint University of Victoria and Vancouver Island Health Authority Research Ethics Sub-committee (#2009-13).

## Results

### Participants

Thirty-two provider participants were recruited across the two communities, eight of which also participated in the debates/discussion groups. Participants roles included: family physicians, hospice physicians, long term care physicians, emergency room physicians, other specialists, home and community care nurses, palliative care nurses, long term care nurses, and pharmacists (Table 2). Some participants had multiple roles (e.g. part time hospice nurse and home and community care nurse) resulting in more roles than participants. When a participant had more than one role they were asked to separate both roles explicitly in their answers.

**Table 2:** Summary of research participants, roles and collective experience

**Study interview summary data**	
Number of Providers Interviewed	32
Physician Participants	13
Nurse Participants	16
Pharmacist Participants	3
Number of Clinical Roles	39
Male Participants	10
Female Participants	22
Average Years Experience in Practice	23.0
Average Years Experience in Community	14.7

### Information continuity

Information continuity was supported in our findings. Participants regularly highlighted challenges in receiving/sharing information as a difficulty in maintaining continuity. As one participant described:

“Sometimes you get the lab and diagnostics [from the ER] and sometimes you have to go and track them down… I obviously prefer to get the information.” (V01)

Several of the gaps were related to cross-organizational information flow:

“The difficulty is that if a person has [private] outpatient labs done, then the outpatient labs do not show up in the [public health authority].” (V06)

Most information flowed from point to point, but some was kept in shared repositories (i.e. charts, electronic records). Some providers felt there was a benefit to having an integrated record across organizations:

“If we had access to all the written information through one [system]… it would be beneficial for our clientele.” (D07)

“There are gaps across the continuum about how those systems and how that information is going to follow a client or patient as they go from home to acute care and back home or to residential care.” (IMIT01)

Finally, considerable time was spent tracking down missing information to ensure continuity:

“The secretary has to phone medical records at the cancer clinic-which she does, multiple time every day… and then you have to wait.” (V07)

### Management continuity

Management continuity was supported by our findings. Participants saw shared care plans as an important part of achieving management continuity (e.g. advance directives) and participants wanted better sharing of those plans across the circle of care and with the patient. However, there were often not good methods to share plans. This impacted many providers, particularly providers of unplanned care, such as emergency room physicians:

“I don’t know how many times I have to make plans for people, first time I meet them. That frustrates me. I point out to the patients-why isn’t there a plan? Why are you directing traffic yourself?” (V13)

Some providers worked diligently to ensure management continuity and shared plans in many ways: to the patient, on paper (which was faxed or shared), through dictation, verbally if urgent, and stored in various charts and records.

“There is a lot of overlap. You write plans in a lot of different places…and then you go back to the office and dictate a letter.” (V07)

### Relationship continuity

Participants confirmed the importance of patient-provider relationships on continuity. This was highlighted in the transition to palliative care at end of life. Participants commented they wanted to stay involved-for relationship continuity:

“Once they are in hospice…I will go in periodically and see the patient, that’s more for morale support and leave the ordering to the hospice physician”; (V01)

“I try to stay involved…I don’t want them to feel abandoned.”; and “I often just like to drop in to say hello. I’ve been involved throughout.” (V02)

The Emergency Department was another place where loss of relationship continuity was felt, particularly in a larger centre, where family physicians were unlikely to be involved in acute care:

“The patient is the loser. The person who really should be there is not there and this is part of this continuity issue. There is nobody in charge anymore.” (V13)

### Provider connectedness

In this study, providers repeatedly discussed the role of relationships between providers in the circle of care. The relationships between providers influenced continuity for their shared patients. This was labeled *Provider Connectedness*. Provider connectedness describes the cohesiveness of the relationships between providers within a circle of care. Providers felt the communication was more effective and thus continuity was easier to ensure when they already had relationships with other providers.

“There is the more social aspect of the continuity of care… speaking as a community family doc, in terms of cohesiveness… cohesiveness of the medical community.” (V03)

Providers who were in physical proximity of each other (e.g. worked on same ward or clinic) or who had cared for many mutual patients over time developed provider connectedness. Several participants had specific activities they did to help maintain these relationships for the benefit of continuity:

“I try to have a good relationship with them [the ER Physicians]… when I go to the hospital, I always go through Emergency…I just say hello to all the guys there, because when I call them, I know who they are. Most of them will call me back.” (V11)

“I’ll just drop by [the GP’s office]…so that he knows me… You want the GP to know at least who you are. Understand that I’m not the home support worker.” (D09)

Those who were physically more isolated did not have as strong provider connectedness. When there were gaps in provider connectedness, trust was not there:

“We don’t know each other and so it becomes very impersonal. With this kind of work, there has to be a really high level of trust between professionals and it’s much harder to trust someone you don’t know than somebody you do know.” (V06)

In the debates participants agreed that this is an important aspect of the circle of care that impacts continuity and that needed to be made explicit. Participants agreed that organizational changes could reduce provider connectedness and had experienced these (see Environmental influences below).

Provider connectedness appeared to have a significant impact on the other types of continuity: information was shared more readily, management was better understood, and providers would ensure providers remained connected to the patient when the providers knew each other.

### Communication patterns related to continuity of care

All types of continuity relied on communication within the circle of care. Participants provided many examples of communication activities. Many activities were person to person (e.g. talking to the patient, faxing the family physician, meeting in the hallway). Some activities are shared with more than one person (e.g. writing a progress note in a shared record). Some are synchronous (e.g. a phone call to notify of an admission) and some asynchronous (e.g. a written prescription).

Through our analysis, we themed these activities based on intention. For example, nurses described shift change handover; physicians described transfers from emergency to ward or from ward to residential care or to hospice; discharge letters were sent to community-based physicians; on-call physicians notified family doctors of patients’ overnight condition and treatment in morning. These separate communication activities share a common intent: to *transfer care* to another provider. Through proper transfers within the circle of care, continuity is maintained. We generalized ten *communication patterns* that were seen across care settings and between different providers (Table 3).

**Table 3:** Summary of the communication patterns related to continuity of care

**Communication patterns**	**Description**	**Examples**
Communicate with Patient/Family	Communicating with the patient to examine the patient’s condition, share information, educate, and to develop a common understanding or plan.	• Patient visits with family physician.
		• Home and Community Care nurse home visit with patient and family.
		• Phone conversation from patient’s daughter with the on call family physician.
		• Medication reconciliation by a pharmacist or nurse.
Request Historical Information (PMHx)^1^	• Seeking additional information from a particular provider, care team, or organization.	• Specialist requests previous blood work from family physician.
		• Hospice requests previous consult letters from Cancer Centre.
Provide Information	Ensuring other providers are aware of current findings and plans by sending information directly to named members of the Circle of Care.	• Follow up letter to family physician from Oncologist on change in chemotherapy.
		• ER Physician note to GP after patient is seen in the Emergency.
		• Home and Community Care Case Manager fax to the family physician to describe care plan.
Document in Shared Record(s)	Documenting findings/plans in a location that is accessible to others (who have access).	• Neurologist documenting in hospital chart.
		• Family physician documenting in Mr. Hart’s long-term care paper chart.
		• Laboratory placing a result into Hospital Information System.
Review Shared Record	Review information shared by other members of the Circle of Care to increase knowledge of patient’s condition.	• Family physician reviews long-term care paper record when rounding on patients.
		• ER Physician reviews hospital information system prior to seeing patient in the ER.
		• Oncologist reviews cancer records (electronic and paper) prior to follow up visit.
		• Pharmacist reviews medication-dispensing history.
Request Advice	Request information and advice about options related to a patient case.	• Call to palliative care hotline to discuss medication options and conversion doses.
		• Call to see what services might be available for particular type of patient.
		• Discuss with radiologist what test is most appropriate for assessing symptom in a patient without disclosing patient name.
Request Assessment/Treatment	Contact another provider to request an action to assess and/or provide treatment recommendations to a patient based on their assessment.	• Family physician consults geriatrics for patient in nursing home to assess behavioural issues.
		• Home and Community Care nurse sends referral to physiotherapy and occupational therapist to assess home safety.
		• ER Physician calls neurology to assess stroke patient.
Order	Request specific activity be delegated to / performed by another provider	• Medication prescription from MD to pharmacist.
		• Home and Community Care nurse delegated medication administration to Community Support Worker.
		• Advance directive from patient.
Transfer Care	Handing off care responsibilities between care providers of a similar capability.	• Nurse handover at shift change.
		• Family physician to family physician transfer when on call.
		• ER physician transfer to family physician admission in hospital once patient is stabilized.
Coordinate as Care Team (i.e. all or part of the Circle of Care)	To review, in real time with more than two individuals, the status and plans for the patient from multiple viewpoints.	• Long-term care case conference.
		• Breast cancer Oncology Rounds.
		• Palliative Care Rounds.
		• Ad hoc meetings between family physician, Home and Community Care nurse and family to discuss patient care or patient prognosis.

^1^*PMHx* Past Medical History.

### Environmental influences

Factors outside the circle of care influence continuity. For example, organizational boundaries and policies can impact continuity. Access to information systems (e.g. due to policies, admitting privileges, cost) impacted continuity. Increased use of casual nurses, walk-in clinics, and hospitalists, were other examples raised by many of the participants as organizational issues that disrupt relationship continuity. Scope of practice, regionalization of services, and regulatory bodies were all provided as examples of environmental includes that impact continuity within the circle.

## Discussion

**Figure 2:**
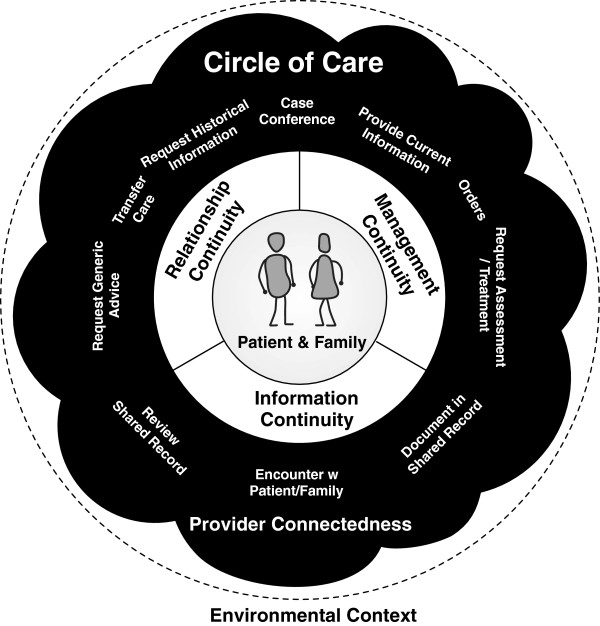
The extended Circle of Care Model of continuity of care.

In this research, we extended the Haggerty and Reid model of continuity to a circle of care-based model of continuity of care (Figure 2). By focusing on the circle of care, we expanded our understanding of how this system has features that support continuity. Taking a systems view allowed us to focus on provider experience of continuity and on systems’ features of continuity that have been less of a focus previously. We have added four elements to the discussion of continuity. Provider connectedness that facilitates achieving continuity between providers. Communication patterns that enable continuity between providers and between providers and the patient. Circles of care that are the systems in which continuity exists and can have characteristics that can impact continuity. Environmental factors that influence the circle of care and continuity but are outside the circle.

Aspects of these elements are included in the discussion of other models. Haggerty and Reid recognize provider experience of continuity includes having information and also “that their care inputs will be recognised and pursued by other providers” [19]. Our extensions have made explicit how they share information and connect with others in the circle. Sparbel and Anderson include system issues [14,15] in their models; however, their systems issues are more consistent with environmental influences in our extension. They did not consider continuity within an explicit system as was done here. By making these aspects explicit, the extended systems based model can support health planners and researchers.

### Implications for health system planners

Health planners can use this extended model to explore changes to enhance continuity. For example, by centering primary care services in patients’ medical homes [39], the nature of the circle changes and may well improve continuity of care. Provider connectedness increases through increased contact between providers who share more patients. Communication patterns are enabled such as: more members of the circle use a shared record, and case conferencing is facilitated by physical proximity. While adopting clinical information systems, health planners can consider how these systems support the communication patterns needed to achieve continuity.

Health planners can use the study methods when exploring issues of continuity. Personas can shape data collection and models can be then explored to assess questions of continuity, such as: Are transfers of care adequately supported? Is a shared record appropriately accessible by members of the circle of care? Do organizational policies (an example of an environment factor that impacts continuity) limit access to information in ways that limit continuity? Does the regionalization of services cause fragmentation in care by expanding the circle of care? Thus planners could improve continuity as they shape policy and practice. That was the intent of the original research from which this extended model was derived [37] and was effective in engaging a multi-disciplinary group to collectively reason about feasible improvements in continuity.

### Future research

This extended model presents new research opportunities at four levels. First, this extension needs to be further tested: it needs to be assessed in other communities. Second, a deeper understanding of provider connectedness is needed, with quantitative measures that can be used to guide healthcare system design. Third, quantification of communication patterns is needed and assessing their effectiveness in enhancing the three aspects of continuity. Fourth, more detailed qualitative and quantitative descriptions of circles of care for patients in different settings/communities will be important. We do not know optimal sizes of the circle for different types of patients. Finally, we need to develop a better understanding of the environmental elements impacting continuity of care. Thus, this extension could inform future work on a range of measurable continuity indices, which are needed [40].

### Limitations

This study was scenario based and focused on complex end of life patients. It did not capture all possible scenarios for end of life care and it may not generalize to other types of care. While two contrasting communities were chosen, external validity of the study is limited as these communities were in the same health jurisdiction. The study was focused on actionable change and may have missed other aspects of continuity. Only one researcher did the interviews and coding. While a supervising committee reviewed the findings, a second independent coding could have improved reliability. Finally, the approach assumed that the two personas had family physicians. The circles of care would be different for people that do not have a family physician.

## Conclusions

This paper presents an extension to the Haggerty and Reid model of continuity of care by considering continuity from a systems perspective. Continuity is achieved through the interactions across the patient’s circle of care. By taking a systems approach, we have extended the Haggerty and Reid model with provider connectedness; ten communication patterns that enable continuity; the circle of care as the system; and the environmental context in which the circle of care operates. Health planners can use this extended model as they consider changes to policy and practice. Researchers can use this extended model as they consider new continuity indices.

## Competing interests

Both authors declared that they have no competing interests.

## Authors’ contributions

Drs. MP and FYL were both involved in the conceptualization of the research study and review of the findings. Dr. MP drafted and was primary author of the paper, with Dr. FYL providing critical revisions of the manuscript. Both authors read and approved the final manuscript.

## Pre-publication history

The pre-publication history for this paper can be accessed here:

http://www.biomedcentral.com/1472-6963/13/309/prepub
